# Exploring the Use of “Nudges” to Improve HIV and Other Sexually Transmitted Infection Testing Among Men Who Have Sex with Men

**DOI:** 10.1007/s10508-022-02321-8

**Published:** 2022-06-16

**Authors:** Ei T. Aung, Christopher K. Fairley, Eric P. F. Chow, David Lee, Kate Maddaford, Rebecca Wigan, Daniel Read, Umar Taj, Ivo Vlaev, Jason J. Ong

**Affiliations:** 1 Melbourne Sexual Health Centre, Alfred Health, 580 Swanston Street, Melbourne, VIC 3053 Australia; 2 Central Clinical School, Faculty of Medicine, Nursing and Health Sciences, Monash University, Melbourne, VIC Australia; 3 Warwick Business School, University of Warwick, Coventry, UK; 4 Centre for Epidemiology and Biostatistics, Melbourne School of Population and Global Health, The University of Melbourne, Melbourne, VIC Australia

**Keywords:** Reminder system, Sexually transmitted infections (STI), Sexually transmitted diseases (STD), Behavioral economics, Men who have sex with men, Sexual orientation

## Abstract

Behavioral economics and its applied branch “nudging” can improve individual choices in various health care settings. However, there is a paucity of research using nudges to improve regular testing for HIV and other sexually transmitted infections (STIs). The study examined which reminder system and message type men who have sex with men (MSM) preferred to remind them to undergo regular 3-monthly HIV and STI testing. A cross-sectional survey study was conducted among MSM attending a sexual health clinic in Melbourne, Australia between 13 January and 5 March 2020, exploring the preferred method of reminder and framing of the message. Descriptive statistics and logistic regression were used to analyze the data. A total of 309 responses were received. The majority of the participants (90%) preferred short messaging service (SMS) as the reminder method for HIV/STI testing compared to other types (e.g., email or instant messaging). More than a third of the participants (45%) showed a preference for a neutrally framed reminder message (Your next check-up is now due. Please phone for an appointment), while one-third (35%) preferred a personalized message (Hi [first name], you are due for your next check-up. Please phone for an appointment). Younger men were more likely to favor positive framed messages than older men who favored neutrally framed messages (*p* < .01). SMS was the preferred reminder method for regular HIV/STI testing. Reminder messages that were neutrally framed, personalized or positive framed messages were preferred over negative or social norm messages.

**Supplementary Information:**

The online version contains supplementary material available at 10.1007/s10508-022-02321-8.

## Introduction

Regular human immunodeficiency virus (HIV) and sexually transmitted infection (STI) screening is important for those at highest risk (e.g., men who have sex with men [MSM] who practice condomless sex, have multiple partners, or use substances before and/or during sex). Several national guidelines recommend that sexually active MSM have an HIV/STI screening every three months (Australasian Society for HIV Medicine, 2018; British Association for Sexual Health and HIV Clinical Effectiveness Group Guidelines, 2015; European Centre for Disease Prevention & Control, 2015; STIs in Gay Men Action Group (STIGMA), 2019). Regular STI screening plays an integral role in controlling the ongoing pandemics of HIV and STIs by diagnosing HIV and STI infections in a timely manner so that early treatment can reduce morbidity and onward transmission (Adam et al., 2014). The Australian Gay Periodic Survey reports an increase in HIV and STI testing from 2016 to 2020, although a decline in HIV testing in the previous 12 months in HIV negative men not on pre-exposure prophylaxis (PrEP) is reported for the same survey duration (Broady et al., 2020). One way to increase early detection of HIV/STI infection with desirable outcomes for the individual and public health is effectively using strategies from behavioral economics.

Behavioral economics, often called “nudging” when applied, is a discipline that applies psychological insights to individual economic decision-making (Thaler & Sunstein, 2009). The decision to obtain an HIV/STI test is an economic one since it involves making trade-offs between benefits (early treatment of STIs) and costs (taking extra time to test, difficulties in accessing clinical services, or test anxiety). Governments have used nudging to improve decision-making in many domains (Behavioural Economics Team of the Australian Government June 2020; Darnton, 2008; Thaler & Sunstein, 2009). A nudge is a change to the choice context made by a choice architect designed to influence behavior in a predictable way that is good for the individual and the wider community (Thaler & Sunstein, 2009). Nudges are attractive policy options because they can be highly effective, maintain the autonomy of those being nudged, and can often be achieved at low cost and with little disruption to existing systems.

One form of nudge is message framing. Messages can be framed so that the same decision or choice can be presented using a positive or negative framing of identical costs or benefits. Due to the principle of loss aversion, this can change the relative attractiveness of the choice options (Kahneman & Tversky, 1979). Although evidence around the framing of health messages has been shown to have an inconsistent effect on health consumers’ behavior (Akl et al., 2011), some studies have demonstrated the impact of positive and negative framing of health care messages was dependent on multiple factors such as health literacy, health behaviors, age-differences, target audience and individuality (Akl et al., 2011; Garcia-Retamero & Galesic, 2010; Rothman et al., 1999; Sherman et al., 2006).

Although we know from behavioral economics that under many circumstances, message framing (i.e., what words are used) and the message medium (i.e., how it is delivered) can influence behavioral outcomes (Farrow et al., 2017; Levin et al., 1998; Martin-Smith et al., 2018; Neuberger & Pabian, 2019; Zhao et al., 2018), few studies have explored the effects of message framing in the sexual health field. These studies have found that message framing impacted sexual decision-making and risk behavior (Camenga et al., 2014; Garcia-Retamero & Galesic, 2010; Kiene et al., 2005). Another message framing used for behavior change is social norms, described as norms or rules that a specific group considers typical or desirable in certain contexts. By influencing the social norms, people can be motivated to follow the norms of groups they follow (Perry et al., 2015; Yamin et al., 2019).

To succeed, message frames (and nudges in general) must find acceptability among the target audience (Reisch & Sunstein, 2016). Acceptability can concern which medium the target audience prefers and what message frames to deploy. Eliciting assessments of acceptability are a key method for customizing nudges. It is also a key way of ensuring that any nudging carried out is ethical, in that it is viewed as a desirable intervention by the relevant audience and less likely to lead to reactance (Arad & Rubinstein, 2018).

This study aimed to understand two behavioral outcomes concerning HIV/STI testing among MSM in a sexual health clinic setting. First, we investigated how people preferred to remind themselves to routinely test for HIV/STI. Second, we tested which additional reminder frames were likely to receive wide support. This study is part of an ongoing project that aims to test whether people who are at higher risk of HIV/STI (as defined by their age and sexual practices: condomless sex, number of sexual partners in the last three months, substance use before and/or during sex) may respond differentially to the framing of the reminder message. Therefore, the data from this study will inform a clinical trial to evaluate whether reframing SMS reminder messages may impact the retesting rates of clients at a single sexual health clinic in Victoria, Australia (ACTRN12619001755123).

## Method

### Participants

Melbourne Sexual Health Centre (MSHC) is a state-funded sexual health clinic in Victoria, Australia, with around 20,000 MSM consultations in 2019. We refer to men who have sex with men only and those who have sex with both men and women as MSM in our study (Aizura et al., 2011). MSHC uses a computer-assisted self-interviewing system (CASI) for all clients to check in on arrival at the clinic, and they were asked with a brief screening questionnaire about their sexual history. Clients are asked if they would like to be reminded of their HIV/STI testing every three months during the routine check-in process. Those who have given permission receive a short message service (SMS) reminder to return for their HIV and STI testing every three months from their first visit at MSHC. It was estimated that 80–90% of men chose a three-monthly reminder return for testing at MSHC (Zou et al., 2013). The standard message they receive is “Your next check-up is now due. Please phone for an appointment,” which is a neutrally-framed message. An anonymous paper-based survey was distributed among MSM aged 18 years and above attending Melbourne Sexual Health Centre (MSHC) between 13 January and 5 March 2020. MSM were identified as men who have sex with men in the previous 12 months on the CASI questionnaire during check-in. This survey aimed to collect data on their challenges in having regular HIV/STI screening.

### Procedure and Measures

Consecutive individuals identified as MSM and 18 years or older were invited to participate in the study by the nurses and doctors during consultations. The survey, a brief participant information sheet and a pencil were packed in an envelope, which nurses and doctors gave out to eligible MSM who gave verbal consent. Each day, the research nurses used computer alerts to remind clinicians and nurses to provide the survey packet to clients who meet the eligibility criteria. The participation rate was unknown as the number of clients who declined to participate was not recorded.

The participants self-administered the surveys, and the completed surveys were then deposited in a secure box in the waiting area at the clinic. Survey data were collected at the end of each day, and data entry was completed by a research assistant RW using REDCap (Research Electronic Data Capture) electronic data capture tools hosted at Monash University. The proportion of men who completed and returned the survey was not known as we presumed once they agreed to participate in the survey, they would return the survey to us. The survey included questions on the frequency of HIV/STI test, reasons for not having a regular three-monthly screening, preferred reminder system, preferred wording on the reminder message, and sexual characteristics such as sexuality, partners in the last three months, taking pre-exposure prophylaxis (PrEP), HIV status, previous STI diagnosis, and reasons for attending MSHC.

The framing of reminder messages was categorized into (1) neutrally framed, (2) personalized, (3) positively framed, (4) negatively framed, and (5) social norms. The framing of messages was populated based on previous research on message framing in health communication (Camenga et al., 2014; Farrow et al., 2017; Kiene et al., 2005; Levin et al., 1998; Martin-Smith et al., 2018; Neuberger & Pabian, 2019; Perry et al., 2015; Yamin et al., 2019; Zhao et al., 2018).

1. Neutrally framed: Your next check-up is now due. Please phone for an appointment (Current reminder method at MSHC).
2. Personalized: Hi (first name), you are due for your next check-up. Please phone for an appointment
3. Social norm: Your next check-up is now due. The majority of people do STI testing on receiving this message. Please phone for an appointment
4. Positive: Your next check-up is now due. To stay healthy, regular testing is recommended. Please phone for an appointment
5. Negative: Your next check-up is now due. Not testing regularly might harm your health. Please phone for an appointment
6. Customized: Your own message. Please specify:

The questionnaire for the survey was included in the supplementary file.

### Statistical Analysis

Descriptive statistics were computed to summarize the participants' sociodemographic characteristics and expressed in percentage. Univariate analysis was conducted on (1) reminder methods and (2) message framing. Independent factors were HIV status, taking PrEP, age, past STI diagnoses, and the number of sexual partners. Analyses were conducted with STATA (StataCorp. 2019. Stata Statistical Software: Release 16. College Station, TX: StataCorp LLC.)

## Results

There were 309 participants who took part in the survey, and two participants were excluded as they did not complete any information on the survey. The mean age of the participants was 32 years (SD = 10). The demographics of the participants are shown in Table 1.

**Table 1:** Demographics of survey participants (*N* = 307)

Demographics	Number of participants	Percentage (%)
*Age*		
Mean, Standard deviation (SD)	32 (10)	
Median, Interquartile range	29 (20–68)	
*Sexual orientation*		
Sex with men only	274	89
Sex with both men and women	33	10
Living with HIV	35	11
Not living with HIV	270	89
Taking PrEP	94	35
Not taking PrEP^a^	175	65
*Sexual partners in last 3 months*		
Median (Interquartile range)	4 (0,40)	
Previously diagnosed with an STI	(*N* = 307)^b^	
Chlamydia	177	68
Gonorrhea	188	72
Syphilis	77	30
*Mycoplasma genitalium*	23	9
Herpes	32	12
Warts	35	13
Hepatitis	9	3
Reasons for visit	(*N* = 307)	
Asymptomatic/regular check-up	152	50
Symptoms	94	30
Contacts of infection	40	13
Treatment/vaccinations	12	4
Others (GP referral/follow up)	9	3
Testing frequency of STI screen (per year)	(*N* = 304)	
Every three months	194	64
Every six months	63	21
Once a year	34	11
Less than once a year	13	4

*HIV* human immunodeficiency virus, *PrEP* pre-exposure prophylaxis for HIV, *STI* sexually transmitted infection

^a^One missing data on PrEP

^b^The total number of MSM with previous STI was more than 307 as an individual can have more than one STI infection in the past

### Barriers to Routine HIV/STI Testing

Half the participants (51%, 158/307) reported having no barriers to routine HIV/STI screening (Table 2). The most commonly reported barriers were related to testing facilities and operating hours. Approximately 21% (64/307) reported long waiting times at the clinic as a barrier, while 14% (43/307) were due to operating hours of the testing facilities, and 12% (37/307) reported long distance to travel to the clinic as a reason. Around 17% (53/307) of participants stated they were forgetful or had no time to get tested due to work. A small proportion of men found it “awkward” (9%, 27/307) or were “ashamed” (6%, 18/307) to get tested. Among 26 participants who specified barriers, the majority described the difficulty and inconvenience of making appointments, especially long waiting times when making phone bookings, and a poor fit between their work commitments and the clinic operating hours. A few reported fear of getting tested or feeling awkward to inform partners if the STI test returned positive.

**Table 2:** Barriers to regular HIV/STI testing for participating MSM and perceived potential barriers for their friends who do not test routinely

Barriers for participants	Number that chose “yes” (*N* = 307)	Percentage %
No difficulties	158	51
Forgetfulness	51	17
Lack of knowledge of testing intervals	23	7
Lack of information on testing centers	7	2
STI testing is not beneficial	0	0
Lack of knowledge of STI symptoms	8	3
Long waiting time at the clinic	64	21
Present to a clinic only when symptoms of STI develop	28	9
Perceptions that STIs are not serious	1	0.3
Social norms-other people are not testing, so why should I?	1	0.3
Feeling awkward getting tested	27	9
Testing procedures intrusive/painful	7	2
Feeling ashamed to get tested	18	6
Unsuitable opening hours at testing facilities	43	14
No time to visit the clinic	53	17
The clinic is too far away	37	12
Staff are rude	4	1
Lack of confidentiality	5	2
Reminder messages too bland or boring	1	0.3
Bad experience with testing in the past	4	1
Other	26	8
Possible barriers for friends who do not test routinely	*N* = 123	%
Stigma/fear/anxiety/embarrassment/shy	44	36
Time factors^a^	32	26
Low risk^b^	30	24
Education^c^	26	21
Laziness/forgetful	14	11
Only test when symptomatic	9	7
Cost	4	3
Needle phobia	3	2
Confidentiality	2	2

The total percentage does not add up to 100% as multiple options were available

^a^Time factors—long wait time, busy with work, long distance to travel to the clinic, limited opening hours

^b^Low risks—being in a stable relationship, considered themselves low risk, use protection, use of PrEP and hence, felt safe

^c^Education—lack of knowledge, awareness of STI, education of STI, not knowing where to access services

Men were also asked why their friends (gay and bisexual men) might not be getting routine HIV/STI testing, and 40% (123/307) reported a range of themes similar to their perceived barriers to routine HIV/STI testing as reported in the previous paragraph. The additional common themes reported were stigma around HIV/STI testing, fear and anxiety of testing, not knowing where or how to get tested (lack of knowledge and awareness), or time factors such as long wait time at clinics, limited opening hours, or a (perhaps mistaken) sense of security among those who were on PrEP or using protection or in a stable relationship from reduced risk of HIV infection. Others cited laziness and forgetfulness, costs of testing or consultations, needle phobia, only testing when symptoms developed and concerns over confidentiality as barriers for their friends to return for HIV/STI testing (Table 2).

### Reminder Method

The great majority of participants (90%, 265/294, 95% CI: 86–93%) reported SMS as their preferred method for receiving a reminder, although a small number (7%, 22/294, 95%CI: 5–11%) preferred email (Table 3). One person preferred to be reminded through the post, and one through smartphone dating apps (e.g., Grindr, Hornet, BlueD) (0.3%, 1/294, 95%CI: 0.02–2%). No one wanted to receive reminders through instant messaging (e.g., WhatsApp, Instagram, Facebook, Snapchat).

**Table 3:** Preferences of reminder methods to return for HIV/STI testing and message framing in reminder text message among survey participants

	*n/N*	Percentage (95% Confidence interval)
Reminder methods	(*N* = 294)	
SMS	265	90% (86–93)
Email	22	7% (5–11)
Postal	1	0.3% (0.02–2)
Dating social app e.g., Grindr, Hornet, BlueD	1	0.3% (0.02–2)
Instant messaging, e.g., WhatsApp, Instagram, Facebook, Snapchat	0	0%
Other	5	(0.6–4)
Reminder messages	(*N* = 307)	% (95% CI)
*Neutrally framed* Your next check-up is now due. Please phone for an appointment	138	45% (39–50)
*Personalized* Hi (first name), you are due for your next check-up. Please phone for an appointment	107	35% (29–40)
*Social norm* Your next check-up is now due. The majority of people do STI testing on receiving this message. Please phone for an appointment	14	5% (3–7)
*Positive* Your next check-up is now due. To stay healthy, regular testing is recommended. Please phone for an appointment	85	28% (23–32)
*Negative* Your next check-up is now due. Not testing regularly might harm your health. Please phone for an appointment	17	6% (3–8)
*Customized* Your own message. Please specify:	14	5% (3–7)

N.B Participants were able to select multiple preferred messages

Dating social app e.g., Grindr, Hornet, BlueD

Instant messaging, e.g., WhatsApp, Instagram, Facebook, Snapchat

Men who chose “other methods” to receive reminders reported their reminder system was through testing regularly with 3-monthly pre-exposure prophylaxis for HIV (PrEP) appointments. They also mentioned that they were reminded by the clinic for regular booked appointments, although they did not mention what reminder system such as SMS or email was used to remind them about the appointment.

### Preferred Message Framing

We presented participants with six message options and asked which messages they would prefer as an SMS message (See Method for message type and Table 3). The participants showed a preference for the neutrally framed reminder message (45%, 138/307, 95%CI: 39–50%), followed by the personalized message (35%, 107/307, 95%CI: 29–40%) and positively framed message (28%, 85/307, 95%CI: 23–32%). Message framing using social norms was the second least preferred message frame (7%, 21/307, 95%CI: 4–10%). The negatively framed message was the least preferred (6%, 17/307, 95%CI: 3–8%). A small number (5%, 14/307, 95%CI: 3–7%) of participants specified their preferred messages, which mainly consisted of normalized STI testing message (social norms), personalized message, and positively framed message. For example, one man suggested using “It's testing time! Own your health & phone to book an appointment today.”

### Analysis

There were no significant associations between reminder methods and age, PrEP use, number of sexual partners and HIV status. Men with previous STI diagnoses were 2.3 times more likely to prefer SMS messaging than men with no previous STI diagnosis (Odds ratio: 2.3, 95% CI: 1.09–4.89, *p* = .03).

There were no significant associations between message framing and PrEP use, HIV status and previous STI diagnosis. Younger men preferred positively framed messages, while older men preferred neutrally framed messages (*p* < .01). Men with fewer sexual partners might prefer positively framed messages (*p* = .04) (Table 4).

**Table 4:** Logistic regression of factors associated with preference for the framing of reminder messages (*N* = 307)

	Reminder message											
	Neutrally framed		Personalized		Positive		Negative		Social Norms		Customized	
	OR (95% CI)	*p*-value	OR (95% CI)	*p*-value	OR (95% CI)	*p*-value	OR (95% CI)	*p*-value	OR (95% CI)	*p*-value	OR (95% CI)	*p*-value
Age (per year increase)	1.04 (1.02–1.07)	< .01	0.98(0.95–1.01)	.12	0.96 (0.93–0.99)	< .01	0.94 (0.87–1.01)	.08	1.03(1.00–1.08)	.15	1.04 (1.00–1.08)	.12
HIV & PrEP												
HIV negative, Not taking PrEP	1 (ref)		1 (ref)		1(ref)		1(ref)		1(ref)		1(ref)	
HIV positive	1.74 (0.82–3.69)	.15	0.49 (0.19–1.25)	.13	0.79 (0.30–2.04)	.63	0.49(0.06–3.89)	.50	1.89(0.37–9.81)	.45	2.93 (0.70–12.37)	.14
HIV negative, taking PrEP	0.84 (0.49–1.45)	.53	0.75 (0.43–1.32)	.32	1.43 (0.81–2.52)	.22	0.69(0.21–2.23)	.54	1.65 (0.49–5.55)	.42	1.65(0.49–5.55)	.42
Past STI diagnosis												
Yes	1.26 (0.64–2.46)	.50	0.87 (0.45–1.69)	.68	0.95 (0.46–1.93)	.88	0.54 (0.17–1.76)	.31	2.32 (0.30–18.28)	.42	1	
No	1 (ref)		1 (ref)		1 (ref)		1 (ref)		1(ref)		1(ref)	
Number of sexual partners	1.01 (1.00–1.05)	.53	1.00 (0.97–1.04)	.83	0.93 (0.88–1.00)	.04	0.97 (0.87–1.08)	.60	1.036 (0.99–1.13)	.10	1.05 (0.99–1.11)	.08

*CI* confidence interval, *OR* odds ratio, *Ref* reference

## Discussion

Our study is one of the few studies to use concepts from behavioral economics to explore the attitudes of MSM on regular HIV/STI testing and improve engagement with health services subsequently (Cassidy et al., 2019; Lim et al., 2012). Our study demonstrates that MSM preferred neutrally framed, personalized or positively framed messages over negatively framed or social norm messages. The majority of the survey participants preferred SMS reminders over other methods such as email or smartphone dating apps regardless of their age.

The current reminder message at MSHC is “Your next check-up is now due. Please phone for an appointment”, which we called the neutrally framed reminder. This is not a bad choice since it was the most popular message of all tested, and men taking the surveys were familiar with the message—about 45% of the participants preferred this message over all the others. The popularity of this message may be due to status-quo bias or exposure effect, i.e., people like things they are familiar with. However, younger men preferred a positively framed message, “Your next check-up is now due. To stay healthy, regular testing is recommended. Please phone for an appointment” more than older men. On the other hand, older men seemed to prefer neutrally framed reminder messages. The findings imply that our current reminder message is an effective option, although future research comparing different messages and men returning for HIV/STI screening would provide a more accurate picture.

Our study shows positively framed and/or personalized messages may also be more effective in targeting MSM, especially using positively framed messages for younger MSM. Studies have reported the impact of framing messages on sexual risk reduction, changing sexual behavior and sexual decision-making (Camenga et al., 2014; Macapagal et al., 2017). Similarly, our study reports a potential role of using different framing messages in targeted groups of MSM, especially in a high-risk group of MSM who would most benefit from regular 3-monthly HIV/STI screening since early detection translates to earlier treatment and reduced transmission. We could apply our study findings and use positively framed reminder messages in younger high-risk MSM and neutrally framed reminder messages in older high-risk MSM to encourage their uptake of regular HIV/STI screening.

Studies have showed age differences in response to framed health messages with findings that older adults might not be influenced by message framing while younger adults might react more adversely to negatively framed messages with a possible theory that loss is not usually expected in younger people (Mikels et al., 2016). If this theory is true, then younger people might prefer positively framed messages. On the other hand, older people may not be as influenced by the framing effect, suggesting that they may prefer things they are familiar with (Mikels et al., 2016). A meta-analysis found that positively framed (gain framed) messages were more effective than negatively framed (loss framed) messages in promoting prevention behaviors (Gallagher & Updegraff, 2011). However, some studies reported older people responding positively to positively framed health messages (Jayanti, 2010). Moreover, only a few studies have examined the effectiveness of neutrally framed messages although those examining the neutrally framed messages did not find superior efficacy compared to other type of messages (Mitchell et al., 2015; Shahrzad Mavandadi et al., 2018). This implies more research is needed to examine if the use of different framing messages by age group would impact on the uptake of HIV/STI screening in MSM.

How we deliver the message is another crucial aspect of a successful health promotion campaign. The delivery method should be easily accessible and serve as an effective reminder to the targeted population. Our study finds SMS was by far the most popular reminder method for all age groups. Despite the widespread use of dating apps and instant messaging apps, very few people chose to be reminded through the apps. The finding is consistent with other studies that showed SMS reminders are an effective method to increase the likelihood of attending clinic appointments, and therefore improve service delivery (Boksmati et al., 2016; Guy et al., 2012; Kannisto et al., 2014). Moreover, the use of SMS messages is not limited as a reminder but also serves as a means to cancel an appointment, which is quick and easy, therefore, the clinic attendees are likely to use the same service again. Additionally, the process will free up more appointments and we can offer to those who want appointments in short notice. Another important insight is that men with a past diagnosis of STI preferred SMS reminders more than those without, which might indicate that high-risk MSM are likely to respond to the use of SMS for regular HIV/STI screening reminders. This study suggests SMS is expected to be the most effective means of communicating with MSM and that resources deployed elsewhere may not be as efficient. Several studies examining SMS in health services support our finding that SMS is a compelling reminder method (Boksmati et al., 2016; Farmer et al., 2014; Guy et al., 2012; Moran et al., 2018).

Perhaps the most crucial principle in nudging behavior is “if you want people to do something, make it easy.” With this in mind, we searched for barriers to getting tested and identified several. Most of these are related to access to health services. These barriers ranged from restricted opening hours, inconvenient locations, long waiting times and the inconvenience of making phone appointments. Some of these barriers are easier (i.e., less costly) to remove than others, but most are potentially remediable. For instance, making appointments via an app or online platform, and offering flexible opening hours are relatively low-cost solutions. We are already using SMS messaging to remind the clinic attendees about their upcoming appointments and regular HIV/STI screening. Some barriers might be difficult to remove, for example, the location of the clinic. For men who reported distance to travel as a barrier, we could consider facilitating telephone consultations with external pathology referral for HIV/STI testing. We can also emphasize that if consumers, such as the market for sexual health services, believe that effort is being made to provide the service they need, rather than the one we want to deliver, they will also recognize the importance of the service and be more inclined to use it.

There are some limitations to this study. First, it was conducted at a sexual health clinic in Victoria; therefore, the results might not be generalizable to MSM not attending a sexual health clinic. Second, there could be social desirability and/or recall bias in reporting previous STIs, testing frequency, and reasons for visits. Third, stated preference may not reflect testing behaviors, and finally, there could be other intrapersonal (one’s own beliefs, values, attitudes, perceptions, and expectations) or other external factors (e.g., restrictions imposed by the COVID-19 pandemic) that influences a participant’s likelihood to participate in regular 3-monthly HIV/STI testing. Future studies should account for these factors when evaluating the impact of the framing effect of reminder messages. Therefore, a future study will prospectively evaluate the effectiveness of the preferred reminder method and framed messages on actual testing behaviors.

Overall, our study shows that MSM preferred an SMS reminder system using neutrally- or personalized or positively framed messages, and an aversion to negatively framed and one appealing to social norms with potential for integration of these findings in improving HIV/STI testing. More accessible options to health services related to sexual health should also be explored.

## Supplementary Information

Below is the link to the electronic supplementary material.

Supplementary file1 (PDF 545 kb)

## Data Availability

The datasets generated during and/or analyzed during the current study are not publicly available due to sensitive nature of information that might be identifiable but are available from the corresponding author on reasonable request.
