# Supplementary material for: Exploring the Use of “Nudges” to Improve HIV and Other Sexually Transmitted Infection Testing Among Men Who Have Sex with Men
Source: Arch Sex Behav. 2022 Jun 16;51(5):2641–50. doi: 10.1007/s10508-022-02321-8 (PMC9202328; doi:10.1007/s10508-022-02321-8)
Supplement: Supplementary file 1 — Supplementary file1 (PDF 545 kb) [file 10508_2022_2321_MOESM1_ESM.pdf]

Reminders to test for Sexually Transmitted Infections

We are inviting you to be a part of this survey because you are a man who has sex with other men. This is a short, anonymous survey to understand how we can help you with routinely testing for sexually transmitted infections (STIs) including HIV. This is completely voluntary and will not affect the care you will receive today. Your response will help us to improve the way we remind people to get regular testing for HIV/STIs.

1) How often do you currently test for HIV/STIs?
   - Every 3 months
   - Every 6 months
   - Once a year
   - Less than once a year

It is recommended that sexually active men who have sex with men should test for HIV/STIs every 3 months (or at least once a year), even if you experience no symptoms.

2) What would make it difficult for you to have routine HIV/STI testing? (tick all that apply)
   1. I have no difficulties
   2. I would forget
   3. I don't know how frequently I need to test for STIs
   4. I don't know where to go to get tested for STIs
   5. I don't think STI testing is beneficial
   6. I don't want to know if I have an STI
   7. The waiting time in the clinic is too long
   8. I don't test if I have no symptoms
   9. I don't think STIs are serious
   10. Other people are not testing so why should I?
   11. I feel awkward getting tested
   12. Some of the testing procedures are intrusive or painful
   13. I feel ashamed getting tested
   14. Testing facilities do not have suitable opening hours
   15. I have no time to visit the clinic
   16. The clinic is too far away
   17. Staff are rude
   18. Lack of confidentiality is problematic
   19. Reminder messages are too bland or boring
   20. I had bad experiences with testing in the past. Please specify_______________
   21. Other_______________

(21)

3) Reflecting on friends of yours that do not test for STIs routinely, what do you think are the reasons why they might not have regular STI testing?

____

____

4) What is your preferred way of receiving a reminder to test?
   1. SMS
   2. Email
   3. Postal letter
   4. Dating social app e.g. Grindr, Hornet, BlueD
   5. Instant messaging e.g. Whatsapp, Instagram, Facebook, Snapchat
   6. Other___________________

5) What type of wording for the reminder message would you prefer? (Tick all that apply)
   1. Your next check-up is now due. Please phone for an appointment
   2. Hi [first name], you are due for your next check-up. Please phone for an appointment
   3. Your next check-up is now due. The majority of people do an STI testing on receiving this message. Please phone for an appointment
   4. Your next check-up is now due. To stay healthy, regular testing is recommended. Please phone for an appointment
   5. Your next check-up is now due. Not testing regularly might harm your health. Please phone for an appointment
   6. Your own message. Please specify____

About you

6) How old are you?_______ years

7) Do you intend to hold on to your current mobile number in the next 12 months?
   - Yes
   - No – I will not be staying in Australia in the next 12 months
   - No – it is a pre-paid mobile number
   - Other_________________________

8) Do you have sex with:
   - Men
   - Women
   - Both

9) How many sexual partners have you had sex with in the last 3 months? __________

10) Do you take HIV pre-exposure prophylaxis (PrEP)?
    - Yes
    - No

11) Are you living with HIV?
    - Yes
    - No

12) Have you been diagnosed with an STI before?
    - Chlamydia
    - Gonorrhoea
    - Syphilis
    - Mycoplasma genitalium
    - Herpes
    - Warts
    - Hepatitis

13) What was the reason for your visit today?
    - I have symptoms
    - I have no symptoms
    - Other _________________________

Thank you for your participation in the survey.
Please drop off your survey in a marked box in the clinic.
